# Process Evaluation of a Digital Smartphone Intervention (URApp) to Support Adherence to Bladder Training in Community-Based Specialist Pediatric Continence Care in England: Mixed Methods Study

**DOI:** 10.2196/95418

**Published:** 2026-10-27

**Authors:** Lucy Beasant, Jenny Ingram, Davina Richardson, Joanna Clothier, Carol Joinson

**Affiliations:** 1 Bristol Medical School, University of Bristol, Canynge Hall, 39 Whatley Road, Bristol, England, BS8 2PS, United Kingdom, 44 7768186367; 2 Bladder & Bowel UK, 10 Priestley Road, Burrows House, Manchester, England, United Kingdom; 3 Department of Paediatric Nephrology, Evelina London Children's Hospital, SE1 7EH, Westminster Bridge Road, London, England, United Kingdom

**Keywords:** adolescents, digital health interventions, behavior change, daytime urinary incontinence, community pediatric continence care, mixed methods, process evaluation, schools, smartphone, standard urotherapy, URApp, urinary bladder

## Abstract

**Background:**

Daytime urinary incontinence is a common childhood condition that can persist into adolescence, and it is associated with psychological distress, social and educational difficulties, and an increased risk of later mental disorders. The co-designed smartphone app “URApp” was developed in partnership with young people aged 10 to 18 years to support their self-management of bladder symptoms, but it is not yet used by community pediatric continence clinics to facilitate ongoing patient bladder training.

**Objective:**

The study aims to understand the barriers and facilitators to embedding a digital health intervention (URApp) designed to aid adherence to bladder training in children and young people with daytime urinary incontinence in community pediatric continence clinics.

**Methods:**

We conducted a mixed methods process evaluation using the Normalization Measure Development (NoMAD) questionnaire at 4 time points to measure behavior change in nursing teams at 6, 12, and 18 weeks. Teams’ responses to questionnaire items were summarized as median scores and plotted at each time point using petal plots. Qualitative data were collected through focus groups and interviews and analyzed, drawing upon thematic analysis.

**Results:**

Twenty pediatric continence nurses from 5 National Health Service community pediatric continence clinics in England were recruited through purposive maximum variation sampling. URApp was well-received, and nurses were positive about introducing it to patients and felt that URApp’s functionality aligned with existing bladder training provided by National Health Service community clinics. Nurses reported that URApp was easy to introduce, age-appropriate for children aged 10 to 18 years, and accessible to patients with neurodivergent conditions. However, community teams found integration into existing care pathways challenging and did not fully use URApp for urotherapy and bladder diary data collection. Teams lacked confidence in changing from paper to electronic bladder diaries, and although they were positive about young people taking ownership of their bladder condition, they struggled to encourage URApp uptake and were concerned about the lack of parental oversight of bladder diary data in URApp. Successful uptake and patient engagement with URApp were also negatively impacted by barriers in the school environment, such as lack of access to toilets and smartphones.

**Conclusions:**

The successful integration of URApp into community pediatric continence clinics requires cultural change in schools regarding toilet access and healthy drinking. Future implementation of URApp into existing clinical care pathways will need to consider how electronic bladder diary data from URApp can be used more effectively by clinical teams, in partnership with patients and their parents/carers to facilitate the shift from analog to a “digital-first health service.”

## Introduction

Daytime urinary incontinence (UI), the involuntary leakage of urine during the day, is a common child health problem that can persist into adolescence. International prevalence estimates of UI vary across studies due to differing definitions, but approximately 5% to 10% of school-aged children experience daytime UI, with prevalence declining to around 1% to 3% during adolescence [1]. Similar patterns have been reported in UK cohort studies, demonstrating that continence problems persist into adolescence for a clinically important minority of young people [2]. There is robust evidence that the prevalence of daytime UI is higher among girls (4.2% of females and 1.3% of males) at the age of 14 years [3-5]. Daytime UI in children and young people (hereafter referred to as young people) leads to psychological distress, social isolation, adverse impacts on education, and negative self-beliefs such as “feeling like an outsider” [6]; young people with UI are at increased risk of mental disorders in early adulthood [7,8]. Most cases of UI in children and adolescents are not due to underlying neurological or anatomical abnormalities, and the causes are unknown [7]. Despite the prevalence and adverse impacts of UI, most young people feel that they are not adequately supported by current services [9-11].

There are huge variations across the United Kingdom in pediatric continence service provision, and demand on these already overstretched services has increased since the COVID-19 pandemic [12]. Bladder training (standard urotherapy) is a behavior modification intervention that aims to correct bladder filling and voiding function [13]. It is an established conservative first-line intervention for UI that is recommended by the National Health Service (NHS) [14], charities supporting people affected by incontinence (ERIC [The Children’s Bladder and Bowel Charity] and Bladder and Bowel, United Kingdom), and the International Children’s Continence Society [15,16]. Bladder training involves establishing a regular schedule of fluid intake and timed toileting to improve the bladder function and reduce UI [13]. Bladder training can be effective, but success strongly depends on self-motivation, commitment, and ongoing clinical support [9,17]. Young people with UI often feel pessimistic about their prognosis and find it challenging to adhere to bladder training [9]. Treatment failure has cost implications for the NHS and threatens the mental well-being and academic attainment of young people [6,7,9,18,19].

Our research with young people with UI found that they have a strong desire to be involved in decisions about their treatment and to feel supported in self-managing their bladder symptoms [9]. In response to this, we co-designed the smartphone app “URApp” in partnership with young people aged 10 to 18 years [20]. URApp’s core functions are based on existing bladder training approaches [13,21] and on empirical evidence about behavior change techniques to improve adherence [22-25]. These include techniques such as goal setting and reviewing, action planning, personalized feedback, and rewards to aid motivation to adhere to bladder training and to help young people work toward a personalized drinking goal. URApp provides discreet reminders for toilet visits and drinking; allows users to record fluid intake, frequency of toilet visits, and urine volume output; and provides charts to track progress, and it includes a diary to record other useful information such as wetting accidents, mood, types of drinks, or stressful events.

In August 2024, URApp was updated to allow users to export a bladder diary to share with their clinic team by text or email; click on *View the App Guide* [20]. Young people involved in the development and formative evaluation of URApp reported that it was acceptable, usable, engaging, and effective in supporting their drinking and toilet schedules, with many requesting to continue using the app beyond the study [26]. Health care professionals working in secondary care settings also reported that URApp was clinically appropriate and aligned with the guidelines for current bladder training and would use it within their own clinics [26].

Despite URApp already being recommended and used by some health care professionals in pediatric continence care [27], it had not yet been evaluated in community pediatric continence care settings. Digital health interventions, including smartphone apps, could facilitate the shifting of care from hospital to community and from analog/paper-based processes by providing continuous digital support, empowering patients to be active participants in their care, and digitizing patient information for health care professionals, in line with the NHS 10-year health plan.

The aim of the current study was to conduct a process evaluation of URApp in pediatric continence clinics to understand how it is embedded into usual care. We gathered evidence to understand the feasibility and practicalities of embedding URApp in this real-world setting, identifying barriers and facilitators to implementation.

## Methods

### Study Design

This process evaluation used a mixed methods approach based on Normalization Process Theory (NPT). NPT is a framework used to guide the implementation and evaluation of complex interventions [28-30] and to understand how staff routinely incorporate a complex intervention into practice in a specific setting [31]. The Normalization Measure Development (NoMAD) questionnaire [32,33] operationalizes NPT across its 4 constructs: coherence, cognitive participation, collective action, and reflexive monitoring. Coherence refers to key stakeholders’ understanding of and opinions about an intervention. Cognitive participation involves stakeholders’ engagement with the intervention. Collective action involves putting the intervention into practice, and reflexive monitoring focuses on stakeholders’ appraisal of the intervention and its effects [28].

### Participant Characteristics

Pediatric continence nurses who introduced bladder training to young people aged 10 years and older in Children’s Community Continence Services were eligible to participate.

### Study Setting, Sample, and Recruitment

Children’s Community Continence Services are Tier 2 community specialist services comprising nurse-led, multidisciplinary teams that provide more specialized assessment and management of bladder and bowel issues in young people. They aim to reduce unnecessary referrals to secondary care. Clinical coapplicants DR and JC assisted with identifying sites in different regions of England serving young people from diverse socioeconomic and ethnic backgrounds. URApp was not routinely embedded within participating Children’s Community Continence Services before the study. As part of this process evaluation, teams were asked to familiarize themselves with URApp and were encouraged to offer it to eligible young people receiving bladder training during the 18-week study period. Decisions about whether and how URApp was used with individual patients remained at the discretion of pediatric continence nurses. Therefore, the study evaluated the implementation and integration of URApp into routine practice rather than testing a mandated implementation protocol.

We recruited Children’s Community Continence Service nurses from 5 centers to provide a purposive maximum variation sample of 20 participants. DR distributed the study advert to the lead nurse at each site via email (Multimedia Appendix 1), who provided consent to be contacted by LB if interested in finding out more about the study. LB emailed the participant information sheet to each interested site and provided further information at an online meeting. NHS staff completed consent forms via REDCap (Vanderbilt University), a secure encrypted web application for building and managing online surveys and databases [34,35]. All participating team members were asked to familiarize themselves with URApp using *View the App Guide* and by downloading URApp onto a mobile device [20].

### Data Collection and Analysis

Staff completed an online NoMAD questionnaire (Multimedia Appendix 2) via REDCap at baseline and 6, 12, and 18 weeks. The NoMAD questionnaire is a set of 20 questions for assessing implementation processes among participants directly involved in implementing complex interventions in health care [32,33]. Staff at each site were also invited to take part in focus groups or interviews. Focus groups and interviews (lasting around 30‐45 min) were conducted online via Teams, audio-recorded with consent from participants, and transcribed, and data were deidentified. A topic guide (Multimedia Appendix 3) was developed in consultation with the clinical coapplicants. The topic guide drew on the NPT theory and had the flexibility to explore teams’ NoMAD questionnaire responses (eg, if there was “low agreement” with NPT subconstructs at a particular site/time point).

Responses to NoMAD items were analyzed descriptively as follows: “strongly agree” and “agree” were interpreted as “high agreement” (5 and 4), “Neither agree nor disagree” as a “neutral” response (3), and “disagree” and “strongly disagree” as “low agreement” (2 and 1). NoMAD item-10 was reverse-coded so that the higher value indicated more agreement for ease of interpretation. Measures of the “normalization” of practice from the NoMAD questionnaires were summarized as median scores and plotted at each time point using petal plots. Higher agreement with NPT subconstructs and better integration of an intervention are characterized by more uniform “petals,” closer to the outer limits of each petal plot (see Multimedia Appendix 4, Figures SA-SE, petal plots). Qualitative data analysis used the NVivo software (version 14; Lumivero) to organize data and drew on reflexive thematic analysis [36] with a deductive approach to explore how data fitted with NPT constructs and subconstructs. NoMAD outputs were cross-referenced with qualitative data to gain an in-depth understanding of the optimization of URApp in community clinic settings.

### Patient and Public Involvement

The original idea for developing URApp came from our previous research with young people with UI, who told us that they needed more support to manage their symptoms [26] and felt they were not adequately supported by current services. URApp was co-designed and iteratively optimized through extensive work with young people with incontinence to ensure it is age-appropriate and meets their needs [26].

### Ethical Considerations

Ethical approval was granted by the University of Bristol Faculty of Health Science Research Ethics Committee (reference 17560) and by the Health Research Authority and Health and Care Research Wales (IRAS project ID 338625, protocol number 2023‐7539).

## Results

### Participant Characteristics

From April to October 2024, 5 Children’s Community Continence Service teams in England were recruited to take part (Table S1 in Multimedia Appendix 5), with participants at each site completing NoMAD questionnaires from June 2024 to January 2025. Most participants were band 6 or above (specialist) pediatric continence nurses (n=14) and had worked in a pediatric continence clinic setting for up to 5 years (n=12; Table S2 in Multimedia Appendix 5). At baseline, half of the participants (n=10) reported that they had “never used” URApp; 8 participants stated that since the study began, they “sometimes” use it with patients, and 1 had started to “routinely use” the app with patients (Table S3 in Multimedia Appendix 5). Only 3 participants responded “never use” when they completed their final NoMAD questionnaire.

### NoMAD Questionnaire Data

Twelve participants completed NoMAD questionnaires at 4 time points: baseline and 6, 12, and 18 weeks (sites A-D). The remaining 7 participants (site E) completed NoMAD questionnaires at baseline and 6 and 12 weeks (Multimedia Appendix 4). Broadly speaking, sites showed collective agreement with “Coherence” NoMAD items at all time points and high levels of neutrality (score of 3, neither agree nor disagree) in relation to cognitive participation, collective action, and reflexive monitoring items. A detailed descriptive analysis of NoMAD questionnaire data, participants’ free-text response data, NoMAD median scores by sites at each time point, and raw NoMAD data is provided in Multimedia Appendix 6.

### Focus Group and Interview Data

Findings from NoMAD questionnaire data were expanded upon during focus group and interview discussions to gain a more in-depth understanding of why teams struggled to integrate URApp. Five focus groups were conducted with 16 participants, and 3 participants took part in one-to-one interviews. One additional participant who did not complete a NoMAD questionnaire participated in a focus group (pediatric continence nurse or nonnurse, band 5 or below), and one participant who did complete NoMAD questionnaires did not take part in a focus group or interview (specialist pediatric continence nurse, band 6 or above). Illustrative themes, corresponding NPT constructs, and quotes can be found in Table 1 and Multimedia Appendix 7.

**Table 1.** Illustrative themes, corresponding Normalization Process Theory (NPT) constructs, and quotes (N=19).

Theme (NPT construct)/subtheme	Supporting quotation
1. URApp has potential (coherence)	
Positive view of app as age appropriate	*it is nice to have something for them I would say, because with all the toileting information, well a lot of it, is based on younger children in a way, and I felt that this was something a bit more relatable to them.* (participant 4, site B-Interview^a^)
Paper bladder diaries not returned	*[bladder diaries] very few and far between they get returned as well, so compliance is an issue with completing charts. That’s why we thought the app would be a bit easier, because they’re constantly on the phone, even parents.* (participant 7, site C)
2. It’s easy to introduce… But (cognitive participation)	
Easy to introduce	*I think it’s easy to talk to them about it. in terms of actually putting it out there for them to try it, that’s quite a straightforward thing to do.* (participant 12, site D)
Signposting to URApp not effective	*Quite often what I find, if I signpost a young person to it, then they, you see them or talk to them again and they haven’t downloaded it or haven’t looked at it.* (participant 19, site E)
Asking young people to download URApp in clinic more effective	*Most of them we get them to download it in clinic. So we know they’ve got it.* (participant 7, site C)
3. Behavior change is hard (collective action)	
Lack of engagement from young people demotivating for staff	*I’m not seeing the massive change I thought I would see, unfortunately, I’ll still be suggesting it.* (participant 15, site E)
Lack of confidence in changing from paper to electronic bladder diaries	*…other concern from parents and carers would be that actually if you’ve got the young person directly collecting the data how is the parent or carer going to monitor that. Whereas when they’ve got a paper diary invariably it goes up on the fridge, or it gets stuck up in their bedroom.* (participant 2, site A)
4. Using URApp in a school environment (reflexive monitoring)	
Impact of school culture on engagement with bladder training	*there are quite a lot of barriers to doing the good toileting and the good drinking in school*. (participant 9, site D)
Lack of toilet access	*Massive issues over toilet access in the school, and drinking. If the education sector got that on-board we probably wouldn’t have a job. they could probably use their phone more than they’re allowed to go to the toilet.* (participant 7, site C)
Not able to access phone at school	*Then there’s been at least four that I’ve spoken to about it, and the person’s just gone, “Oh they’re not allowed their mobile phones, they’re not allowed the phones in school.” when they can’t use it in school they’re very much like no, can’t use this phone, and a little bit dismissive about it.* (participant 11, site D)
More usable bladder diary data with URApp	*that [neurodivergent male] I said, you know, normally you sit here and you shrug and you don’t know, you don’t know. But we’ve got that phone now, and I’ve seen more data now than I ever have done. I was surprised he was doing it. So he wasn’t one I thought, actually, you’re going to just download it and off you’re going to go because that’s normally what happens. But now I bought him back sort of the, I think two months later. Yeah. and we’ve got some data.* (participant 6, Site B-Interview^a^)

a Indicates interview, all other participants took part in focus groups.

### Thematic Analysis With Links to Core NPT Constructs

#### Theme 1: URApp Has Potential (Coherence—Understanding and Opinion of the Intervention)

Although some team members were aware of URApp and other apps that could be used to support hydration and set reminders, none were actively using URApp as part of their clinical practice prior to their involvement in the study. All team members were able to download URApp onto a phone and were enthusiastic about the prospect of introducing it to patients. Teams had a positive view of app functionality (internalization) and felt that URApp had the potential to improve young people’s engagement with bladder training techniques (internalization). All discussed ways in which URApp could be integrated into clinical practice to support diagnosis, by recording fluid input and output, and to aid adherence to ongoing bladder training through drink and toilet reminders and working toward a drinking goal (communal specification).

Recording data digitally in URApp differed from teams’ usual practice of asking families to record fluid input/output using paper bladder diaries (differentiation). All teams reflected on the fact that tracking young people’s progress using paper-based bladder diaries was not effective because families were either not completing them accurately or frequently did not return them at all (individual specification). Teams could see how URApp reminder functions were similar to their past use of basic alarms to remind young people to use the toilet and/or drink; however, all acknowledged that using a digital app with all these functions was potentially more appealing and accessible for young people and inclusive in terms of neurodiversity (individual specification). Because URApp was a new process, 2 teams acknowledged that they sometimes forgot to discuss it with eligible patients, which had hindered its integration. Team members began to discuss other ways in which URApp could be integrated into their current practice, including supporting young people with an overactive bladder and bowel issues, enabling them to record and share when relevant symptoms were occurring via the “daily diary” function (eg, damp pants and tummy ache) and by allowing young people to develop their awareness of the need to drink and use the toilet regularly, despite not being able to use URApp in school (individual specification).

#### Theme 2: It’s Easy to Introduce… But (Cognitive Participation—Engagement With the Intervention)

All sites reported that URApp was easy to introduce because it closely aligned with the bladder training information they would routinely provide (legitimation). Nurses enjoyed introducing URApp because it was more engaging than paper-based bladder diaries, and most stated that they would continue to introduce the app in the future (activation). Nurses began by signposting families to the URApp website and asked young people to download the app in their own time at home due to limited time during clinic appointments. However, at their next clinic appointment, there were reports of young people having forgotten to download URApp. Those who were not already doing so began to ask patients to download URApp onto their phones during the clinic appointment (legitimation). This was viewed by many as a key initial step in engaging young people and ensuring they did not simply forget to download the app when they got home. Across sites, participants identified practical strategies that appeared to support the introduction and use of URApp in routine care. These strategies were subsequently refined with input from the clinical coauthors (DR and JC) to produce a set of suggested best practice recommendations (Textbox 1). In addition to introducing URApp and explaining how it could be used to support bladder training techniques, 2 sites also made changes to standard team templates, such as including the URApp website link in letters sent to parents/carers after their child’s clinical appointment (site E) or developing a leaflet with the URApp link and logo (site C), to display in waiting areas for families to take away (activation). Two further sites stated that they would consider adding URApp to team templates in the future (sites A and D).

Textbox 1. **Best practice when using URApp in Children’s Community Continence Services.**

1. Ask young person to download URApp onto their phone in their clinic appointment
2. Send URApp weblink in the clinic letter after the initial assessment so that parent/carers also have access
3. Integrate URApp into a new or existing patient care plan
4. Show young person how to record fluid intake and urine output using the “volume” function in URApp
5. Use of URApp to collect volumes of drinking and voiding and data prior to clinic visits/at intervals for 48-hour periods as an electronic bladder diary
6. Ongoing use of URApp to support bladder training—use over a weekend, during school holidays
7. Provide a National Health Service email address so that young person or parent/carer can record/email fluid intake and urine output data to the clinical team

Although one person at each site acted as a “site contact” with the URApp research team, this person had limited time to act as a “champion” for URApp in terms of integrating it more widely into team processes (initiation). At the 18-week point, site D was still “neutral” in relation to NoMAD item 5: “*‘*there are key people who drive URApp forward and get others involved.” Although, according to NoMAD questionnaires, most stated they were open to working with colleagues in new ways to use URApp, engagement with the intervention did not progress to discussions at team meetings about more structured integration of URApp into existing care pathways (enrollment). Teams felt that training and support in the form of a QR code and animation would be useful to facilitate the introduction and ongoing use of URApp. The development of an animation was discussed as something that young people, parents, and staff might use collectively, highlighting key app functions (such as downloading data) and providing useful examples about why certain aspects of bladder training are important. Despite team members liking URApp functionality, believing that it has potential benefits for use in the community clinic setting (coherence), and enjoying introducing it to families (cognitive participation), teams reported poor uptake and lack of long-term engagement with the app from young people. This in turn impacted how teams engaged with URApp in the long term (collective action).

#### Theme 3: Behavior Change Is Hard (Collective Action—Putting the Intervention Into Practice)

Teams were positive about the theory and behavior change techniques that URApp was based on but found these hard to implement in practice. When nurses reengaged with young people who had been introduced to URApp but not downloaded it or had downloaded it but had not continued to use it in the long term, it was demotivating for staff who had initially thought that introducing URApp to patients would motivate young people to engage with bladder training (lack of relational integration, NoMAD item 11). Although nurses discussed more examples of feedback about URApp from female patients, they did not report any specific gender differences in relation to URApp uptake or use; therefore, this may have been due to the fact that the prevalence of daytime UI is higher among females. Some commented that URApp was appropriate for and liked by patients with neurodivergent conditions who prioritized routines or needed reminders in relation to toileting (interactional workability), while others reflected that older teenagers were not engaging with URApp. However, some highlighted that these were often patients who had been managing their condition for a long period of time and had not previously engaged with bladder training. Others felt that younger teenagers liked the rewards (eg, streaks/trophies) or were motivated by the novelty of having access to a smartphone to engage with URApp.

Although nurses discussed actively trying to encourage young people to take ownership of their bladder training, they were doubtful that young people would fully engage with data capture in URApp without monitoring and input from a parent/carer (skill set workability, NoMAD item 12). The strategy of asking young people to use URApp at their initial appointment and to remind or show their nurse how they had used it at their next clinic appointment (which could be 3 or more months later) was also not successful. One focus group highlighted that young people who volunteer to participate in co-creation research (ie, those who codeveloped URApp) may be more motivated than the patients they typically see in their day-to-day clinical practice. Parents/carers were described by all teams as leading or being actively involved in the management of their child’s bladder training; particularly, in relation to recording fluid input/output using a paper-based bladder diary for 2 to 3 days prior to their child attending an initial assessment appointment. Some suggested that URApp should allow a shared parent/child login or a secondary parent/carer URApp platform to minimize these issues.

Although parents/carers played a key role in their child’s engagement with bladder training, one team discussed instances where they encouraged parents/carers to step back and allow their child to take the lead, highlighting the difficulties faced by community teams balancing clinical recommendations with parent/carer preferences and young people’s autonomy. Patients who were proactive and engaged with bladder training were described as making quicker progress in terms of symptom improvement, but teams also reflected on some families’ broader views that medication would be more effective than bladder training. There were also reports of some follow-up appointments being conducted remotely via a speaker or video phone call without the child/young person present. Because the child/young person was not present in these instances, nurses would be unable to discuss URApp since the parent would not have access to their child’s URApp data on their smartphone (lack of interactional workability, NoMAD item 9).

Despite all sites reporting low engagement from patients and their parents/carers when using paper bladder diaries to gather data, several nurses stated that they would not feel confident collecting these data using URApp, and teams did not engage with the data export function available in URApp (lack of contextual integration, NoMAD item 14). Most did not recall that URApp allows young people to measure the volume (in mL) of their fluid intake and urine output, and all reported that the URApp data export function was not used by young people (lack of skill set workability, NoMAD item 13). Currently, URApp does not automatically share bladder diary data with community clinical teams, but young people can download data and send it by email. Teams generally viewed URApp data on the young person’s phone during clinic appointments. None of the teams asked young people to download or email their URApp data for review during the consultation or for inclusion in the patient’s notes (lack of contextual integration, NoMAD item 14).

Although all teams reported that some young people still lacked motivation, they also acknowledged that “it takes a while for parents and children to engage” (P4B) in bladder training. Staff reflected on the timing of NoMAD questionnaires at 6 and 12 weeks, as they noted that patients’ next appointment could be 3 or more months after the appointment in which they had been introduced to URApp. Therefore, nurses may not yet have reviewed a patient who was actively using URApp, potentially resulting in neutral responses to “Collective action” NoMAD items. Nurses also commented that the independent use of URApp for recording input/output over a 3-month period without clinical support would not be manageable for many young people who were typically only asked to record their fluid intake and output for a fixed period (eg, a 2‐3 days paper-based bladder diary). Teams found the integration of URApp into existing care pathways challenging and lacked confidence in changing from paper to electronic bladder diaries. Although they were positive about young people taking ownership of their bladder condition, they were concerned about the lack of parental oversight of bladder diary data in URApp (item 11: Relational integration, item 12: Skill set workability).

#### Theme 4: Using URApp in a School Environment (Reflexive Monitoring—Appraisal of the Intervention)

Nurses’ appraisal of URApp as an effective way of collecting data going forward was also based on reports from young people that they were unable to use URApp effectively in their school environment (a lack of effective external systematization). Patients who were engaging with URApp were largely doing so outside of school, either at weekends or because they were not currently attending school. Nurses were frustrated when patients reported restrictive school toilet policies and inadequate or unpleasant toilet facilities that did not promote healthy bladder and bowel habits or did not enable bladder training adherence. Inadequate toilet facilities resulted in young people with bladder conditions not wanting to drink fluids at regular intervals so that they did not need to use the toilet during the school day. Young people also reported significant issues with some school toilets being locked during lesson time and only accessible during breaks, when significant queues formed. There were also reports of teachers restricting access during lesson time, only allowing those with a medical or toilet pass to leave the classroom, and the use of chaperones. Young people reported that they did not want to use a medical/toilet pass to gain access to toilets during lesson time because this might highlight their condition and lead to unwanted questions from peers.

Nurses highlighted that many schools now have strict phone use policies; they either do not allow young people to use their smartphones at all during school hours or they do not allow phone use during lessons (lack of positive communal appraisal of URApp). Since toilets in some schools were only open during breaks and at lunchtime, pupils drank water less to avoid the need to use the toilet, and there were also reports of young people having limited access to drinking water during lessons (eg, not being allowed to drink water at their desks). Despite these challenges, all teams were very positive about continuing to introduce URApp to young people, and NoMAD questionnaires showed agreement from nurses on how they could modify and work with URApp in the future (item 20: reconfiguration). One team was moving toward patient-initiated follow-up and felt that URApp could potentially fit well with this new approach. Alternatives to using URApp in the school environment were suggested, such as advising young people to focus on using URApp at weekends when they have less structure in their day and struggle to remember to drink regularly and use the toilet. Individual appraisal, in terms of “valuing the effects that URApp has had on my work,” was apparent when a team member had a direct experience of seeing data output from URApp when it was used effectively by patients, particularly by those who had not previously engaged with bladder training.

## Discussion

### Principal Findings

This is the first study to explore facilitators and barriers to embedding a digital health intervention (URApp) in Children’s Community Continence Services in England. We used a mixed methods approach combining results from the NoMAD questionnaire with qualitative data from interviews/focus groups. The main findings of this study were that URApp’s functionality, alignment with bladder training techniques, age appropriateness, and accessibility (eg, supporting young people with neurodivergent conditions) facilitated staff engagement with URApp. Although nurses found URApp engaging and easy to introduce, they reported broader challenges relating to young people’s engagement with bladder training. School toilet access policies were reported as a key barrier to successful engagement with bladder training. Nurses did not engage with the bladder diary data export function in URApp and had concerns about relying on young people to self-manage their bladder training and digital data without access being granted to parents/carers. We found that nurses acknowledged the importance of young people taking ownership of managing their bladder symptoms; however, they also stressed their belief that successful management should be part of a shared decision-making model that includes positive support from parents/carers. Although our sample was not large enough to draw firm conclusions, our findings tentatively suggest that engagement with URApp may vary by age, with younger children appearing more responsive to app-based motivational features than older teenagers. This may reflect age-related differences in motivation and preferences but could also be influenced by changing attitudes toward smartphone use among young people, including increasing awareness of screen time and digital well-being. Policies limiting mobile phone use in schools were major barriers to engagement with URApp. These factors warrant further investigation when optimizing and implementing digital bladder training interventions.

### Strengths and Limitations

This is the first study in this area to use a process evaluation approach guided by NPT; we provide valuable insights into the challenges and facilitators of embedding a digital health intervention in community pediatric continence services. Using a mixed methods approach enabled us to capture behavior change over an 18-week period and to gain an in-depth understanding of current clinical practice. Challenges included clinical teams’ resistance to changing usual practice (eg, paper-based bladder diaries) and school-based barriers that prevented young people’s engagement with bladder training (policies relating to toilet access and mobile phone use). Our study included Children’s Community Continence Service teams in different geographical regions of England, serving rural and urban populations, but transferability of our findings is limited by the small sample size and the small number of teams involved in the study. We aimed to recruit nurses with varying levels of experience, but our sample mainly comprised nurses with up to 5 years of experience in Children’s Community Continence Services. We were only able to capture behavior change in the clinical teams over a relatively short period of time (at baseline and 6, 12, and 18 wk). Constraints included the short period of funding for the study (1 y) and the length of time taken to gain capacity and capability in the 5 participating NHS sites. Data collection time points did not align well with the time period (approximately 3 months) between the initial clinic appointment, in which URApp was introduced, and routine follow-up appointments. Some team members reported that they had not yet seen patients who had previously been introduced to URApp at a follow-up appointment. Although young people were involved throughout the development and optimization of URApp [26], patient and public involvement was not incorporated into the design, conduct, or interpretation of this process evaluation. The absence of young people and parents/carers as study participants may have limited our understanding of implementation barriers and facilitators from the perspective of end users.

### Links With the Existing Literature

Empirical evidence indicates that bladder training can be effective [13,37,38]; however, the success of bladder training depends on motivation and continued engagement [39,40]. There is evidence that digital health interventions can support pediatric bladder training [26,41,42], but barriers include individuals’ beliefs about the effectiveness of conservative interventions, clinicians’ resistance to adopting new approaches, and environmental barriers.

### Engagement With Conservative Interventions

One of our key findings was that children and young people experienced difficulties engaging with bladder training because of concerns about standing out from their peers at school and dissatisfaction with school toilet facilities. This aligns with findings from a European focus group study of children aged 9 to 13 years, which identified fear of attracting unwanted attention and dissatisfaction with school toilets as important influences on engagement with bladder training [39]. We also found that participants described frustration with symptom relapse and a lack of long-term engagement with URApp. These findings are consistent with previous UK research in which young people reported frustration with bladder training due to treatment failure and relapse, alongside disengagement from treatment following unsuccessful symptom management [9]. Participants described how school-related barriers could limit adherence to bladder training, which may contribute to perceptions among parents and young people that medication is a more practical treatment option. Similar barriers have been reported in studies of children’s bladder health in school settings [43]. However, evidence from adult populations suggests that bladder training may be as effective as, or more effective than, anticholinergic medication for improving bladder symptoms [44]; therefore, further research is needed in pediatric populations.

### Resistance to Adopting Digital Systems

Although health care professionals recognized the potential advantages of electronic bladder diaries, many remained reluctant to replace established paper-based approaches. This finding is consistent with implementation research showing that the adoption of digital innovations depends on staff confidence, organizational support, and the presence of local champions who facilitate new ways of working [45-47]. Evidence from mobile health research similarly highlights the important role of health care professionals in supporting engagement with digital interventions [48]. Our findings therefore suggest that implementation efforts should focus not only on patient engagement but also on enhancing health care professionals’ confidence and readiness to adopt digital continence technologies.

### Environmental Barriers

Although a recent systematic review reported that mobile apps for urological conditions can improve the quality of life and symptoms in adults through real-time data capture and self-monitoring [49], our findings suggest that the benefits of digital bladder training interventions may be limited by the school environment. Participants described school policies that limit access to toilets (only during morning/afternoon break or at lunchtime) and reported that toilet facilities were inadequate and unhygienic. These findings are consistent with previous research demonstrating that school environments can hinder healthy toileting and drinking behaviors [6,50-53] and are further supported by a recent systematic review, showing that restricted toilet access, poor toilet conditions, and privacy concerns represent significant barriers to healthy toileting behaviors in a school environment [54]. These findings highlight the need for greater awareness among school staff of the ongoing toileting requirements associated with bladder training, particularly in school environments where toilet access is closely regulated [55].

Participants also highlighted restricted access to mobile phones during the school day as a potential barrier to engagement with URApp. This challenge may become increasingly important as schools adopt stricter policies regarding phone use [56,57]. Although smartphone ownership among young people is high [58], more than half of children aged 8 to 17 years are not permitted to use their phones during school hours [59]. While UK guidance emphasizes schools’ responsibilities to support pupils with medical conditions, including access to drinking water and toilet breaks when needed, it does not address the use of digital health tools to support self-management [55,57]. Given growing evidence that mobile health interventions can improve adherence and self-management across a range of health conditions [60], further research is needed to determine how school phone policies can support equitable access to digital health interventions for young people with medical conditions.

### Implications for Policy, Practice, and Future Research

The successful implementation of digital bladder training interventions requires action at multiple levels. Within Children’s Community Continence Services, efforts should focus on increasing NHS staff confidence in digital tools and integrating electronic bladder diary data into routine practice. Although health care professionals recognized the potential value of URApp, reluctance to replace established paper-based approaches suggests that staff training, organizational support, and local implementation champions may be important for successful adoption [45,61]. Our findings also highlight the importance of involving young people, parents/carers, and health care professionals in shared decision-making. Previous research found that young people wished to take ownership of their continence management and viewed smartphone apps as a supportive tool for self-management [26]. Future research should explore how shared decision-making influences engagement with digital interventions and adherence to bladder training.

Beyond health care settings, participants in this study consistently identified the school environment as a major barrier to bladder training adherence. Schools should be supported to develop policies that balance educational priorities with pupils’ needs for regular toilet access and hydration [6,52,54]. In addition, schools should consider how digital health technologies can be accommodated for pupils who rely on them to manage long-term health conditions [55,57]. As schools increasingly adopt restrictions on mobile phone use, alternative implementation strategies may be required, including negotiating health-related exemptions, permitting their use during school holidays, or delaying data entry outside school hours. More broadly, our findings highlight the importance of considering organizational, educational, and policy contexts when implementing digital continence interventions.

### Conclusions

Using URApp in community clinics has the potential to align pediatric continence care with the NHS 10-year health plan [62] by focusing on early intervention in a community setting and using digital technology to capture and store data. URApp is free to download, available for both iOS and Android phones, and has the potential to empower young people to self-manage their symptoms between clinic appointments. Digitizing bladder diaries may lead to improved treatment outcomes, improved patient experience, and reduced barriers to accessing health care. Future implementation of URApp into existing clinical care pathways will need to consider how electronic bladder diary data from URApp can be used more effectively by clinical teams, in partnership with patients and their parents/carers.

## Supplementary material

10.2196/95418 Multimedia Appendix 1 Study advert and email subject.

10.2196/95418 Multimedia Appendix 2 Normalization Measure Development Questionnaire.

10.2196/95418 Multimedia Appendix 3 Topic guide.

10.2196/95418 Multimedia Appendix 4 Petal plots.

10.2196/95418 Multimedia Appendix 5 Participant characteristics.

10.2196/95418 Multimedia Appendix 6 Normalization Measure Development Questionnaire data descriptive analysis.

10.2196/95418 Multimedia Appendix 7 Illustrative themes and quotes.

## Abbreviations

ERIC: The Children’s Bladder and Bowel Charity
NHS: National Health Service
NoMAD: Normalization Measure Development
NPT: Normalization Process Theory
UI: urinary incontinence
