# Supplementary material for: Process Evaluation of a Digital Smartphone Intervention (URApp) to Support Adherence to Bladder Training in Community-Based Specialist Pediatric Continence Care in England: Mixed Methods Study
Source: JMIR Nurs. 2026 Oct 27;9:e95418. doi: 10.2196/95418 (PMC13712171; doi:10.2196/95418)
Supplement: Supplementary file 1 [file nursing-v9-e95418-s001.docx]

**Multimedia Appendix 1: Study advert & Email subject: Evaluating a smartphone app to support bladder training**

Dear [insert name],

We’d like to invite you to help us evaluate a smartphone app, URApp, (<https://urapp.org.uk/>) which is designed to aid adherence to bladder training. URApp is specially designed for young people aged 10-19 years with daytime wetting. We are conducting a study to examine how URApp could be embedded within paediatric continence clinics.

**Who are we inviting?** Clinicians working in paediatric continence clinics in England and Wales. We are looking for up to four members of staff at each clinic to participate in the study.

**What does it involve?** We will invite you to complete a brief (five minute) online questionnaire at four timepoints during 2024 to gather your views of URApp and to allow us to see if your views change over time. We might also invite you to participate in an interview or focus group lasting 30-45 minutes.

**When does it start?** We will ask you to complete a baseline questionnaire as soon as your site joins the study, and at 2-3 further timepoints up until December 2024. The interviews or focus group are likely to take place in November/December 2024.

To compensate for NHS staff time on the study, we will provide up to £800 to each participating NHS trust’s research fund. This comprises £200 for each timepoint of participation.

**If you are interested in taking part, please contact Dr Lucy Beasant (**[**Lucy.Beasant@bristol.ac.uk**](mailto:Lucy.Beasant@bristol.ac.uk)**), Senior Research Associate, or Professor Carol Joinson (**[**Carol.Joinson@bristol.ac.uk**](mailto:Carol.Joinson@bristol.ac.uk)**), Principal Investigator**
