# Supplementary material for: Process Evaluation of a Digital Smartphone Intervention (URApp) to Support Adherence to Bladder Training in Community-Based Specialist Pediatric Continence Care in England: Mixed Methods Study
Source: JMIR Nurs. 2026 Oct 27;9:e95418. doi: 10.2196/95418 (PMC13712171; doi:10.2196/95418)
Supplement: Supplementary file 2 [file nursing-v9-e95418-s002.docx]

**Multimedia Appendix 2: NoMAD questionnaire**

| **Part A: About you** | |
| --- | --- |
| 1.**How many years have you worked in a paediatric continence clinic setting?**   Less than one year; 1-2 years; 3-5 years; 6-10 years; 11-15 years; More than 15 years   2.**How would you describe your professional job category? Paediatric continence nurse (Band 5 or below)**   Paediatric continence nurse (Band 5 or below), Specialist paediatric continence nurse (Band 6 or above), Other- please specify if the above categories do not capture your role   3.**Do you currently use URApp with patients in your clinic?**   Use routinely; Use sometimes; Never use   4.**Please describe how/who you sometimes use URApp with:**   Free text box | |
| **Part B: Questions about URApp (**NPT Constructs, sub-constructs and questions) | |
| **Coherence:**  Understanding and opinion of the intervention  *(NoMAD items 1-4)* | 1.**Differentiation:** I can see how [URApp](https://urapp.org.uk/) differs from usual ways of working. 2.**Communal specification:** Staff in this organisation have a shared understanding of the purpose of [URApp](https://urapp.org.uk/). 3.**Individual specification:** I understand how [URApp](https://urapp.org.uk/) affects the nature of my own work. 4.**Internalization:**   I can see the potential value of [URApp](https://urapp.org.uk/) for my work. |
| **Cognitive participation:**  Engagement with the intervention  *(NoMAD items 5-8)* | 5.**Initiation:** There are key people who drive [URApp](https://urapp.org.uk/) forward and get others involved. 6.**Legitimation:** I believe that using [URApp](https://urapp.org.uk/) is a legitimate part of my role. 7.**Enrolment:** I'm open to working with colleagues in new ways to use [URApp](https://urapp.org.uk/). 8.**Activation:** I will continue to support [URApp](https://urapp.org.uk/). |
| **Collective action:**  Putting the intervention into operation  *(NoMAD items 9-15)* | 9.**Interactional workability:** I can easily integrate [URApp](https://urapp.org.uk/) into my existing work. 10.**Relational integration:** [URApp](https://urapp.org.uk/) disrupts working relationships. 11.**Relational integration:** I have confidence in other people's ability to use [URApp](https://urapp.org.uk/). 12.**Skill set workability:** Work is assigned to those with skills appropriate to [URApp](https://urapp.org.uk/). 13.**Skill set workability:** Sufficient training is provided to enable staff to implement [URApp](https://urapp.org.uk/). 14.**Contextual Integration:** Sufficient resources are available to support [URApp](https://urapp.org.uk/). 15.**Contextual Integration:** Management adequately supports [URApp](https://urapp.org.uk/). |
| **Reflexive monitoring:**  Appraisal of the intervention  *(NoMAD items 16-20)* | 16.**Systemization:**  I am aware of reports about the effects of [URApp](https://urapp.org.uk/). 17.**Communal appraisal:** The staff agree that [URApp](https://urapp.org.uk/) is worthwhile. 18.**Individual appraisal:** I value the effects that [URApp](https://urapp.org.uk/) has had on my work. 19.**Reconfiguration:** Feedback about [URApp](https://urapp.org.uk/) can be used to improve it in the future. 20.**Reconfiguration:** I can modify how I work with [URApp](https://urapp.org.uk/). |
