# Supplementary material for: Process Evaluation of a Digital Smartphone Intervention (URApp) to Support Adherence to Bladder Training in Community-Based Specialist Pediatric Continence Care in England: Mixed Methods Study
Source: JMIR Nurs. 2026 Oct 27;9:e95418. doi: 10.2196/95418 (PMC13712171; doi:10.2196/95418)
Supplement: Supplementary file 3 [file nursing-v9-e95418-s003.docx]

**Multimedia Appendix 3: Topic Guide**

**Topic Guide**

Objectives: We would like to understand how clinical teams have integrated URApp into their day-to-day clinical practice and to explore your views and experiences of URApp.

**Introductions**

1.	Current work patterns

2.	Care pathway – how will patients have made their way to your service e.g. via GP referral?

3.	Will patient[s] have already completed a 2-3 day paper bladder diary and returned it to you before the meeting where you introduce URApp?

**Coherence: Understanding and opinion of the intervention**

4.	What are your general views on introducing URApp to patients?

[Prompts: Was this a completely new practice in your unit, had any patients mentioned/used URApp before the study started?]

5.	How do you introduce/use URApp during the initial/follow-up appt[s]?

[Prompts – use of URApp website, URApp on HPs phone, does young person download during appt or after?]

**Cognitive participation: Engagement with the intervention**

6.	What kind of barriers have been encountered to embedding URApp?

[Prompts – clinic specific / child specific / environment specific]

7.	Has anything helped when embedding URApp?

[Prompts – clinic specific / child specific / environment specific]

**Collective action: Appraisal of the intervention**

8.	How has your team incorporated URApp into day-to-day practice? [Prompts - individually and collectively as a team?]

9.	Do you have enough support to use URApp? [Prompts - time/resources/ management]

10.	Does the website/using URApp provide enough background/training to enable you to use URApp with patients? [Prompts - Any potential issues with scaling up? suggestions for improving the URApp website?]

**Reflective monitoring: assessing the effectiveness of URApp**

11.	What have patients fed back in relation to using URApp? [Prompts – effectiveness, likes, dislikes, usability, additional functions/improvements?]

12.	Any staff feedback on URApp? [Prompts – effectiveness, likes, dislikes, usability, additional functions/improvements?]

13.	Do you think that URApp will become a routine part of planning care with patients? [Prompts - how/why/why not?]

14.	Any further comments or thoughts about what we discussed?

Thank you for your time.

[The topic guide is flexible and may be amended in response to NoMAD questionnaire data].
