# Supplementary material for: Process Evaluation of a Digital Smartphone Intervention (URApp) to Support Adherence to Bladder Training in Community-Based Specialist Pediatric Continence Care in England: Mixed Methods Study
Source: JMIR Nurs. 2026 Oct 27;9:e95418. doi: 10.2196/95418 (PMC13712171; doi:10.2196/95418)
Supplement: Supplementary file 4 [file nursing-v9-e95418-s004.docx]

**Multimedia Appendix 4: Figures A-E petal plots**

**Figure A: Petal chart showing median scores for the 20 NoMAD questions at 4 timepoints (Site A).**

**Figure B: Petal chart showing median scores for the 20 NoMAD questions at 4 timepoints (Site B).**

**Figure C: Petal chart showing median scores for the 20 NoMAD questions at 4 timepoints (Site C).**

**Figure D: Petal chart showing median scores for the 20 NoMAD questions at 4 timepoints (Site D).**

**Figure E: Petal chart showing median scores for the 20 NoMAD questions at 3 timepoints (Site E).**
