# Supplementary material for: Process Evaluation of a Digital Smartphone Intervention (URApp) to Support Adherence to Bladder Training in Community-Based Specialist Pediatric Continence Care in England: Mixed Methods Study
Source: JMIR Nurs. 2026 Oct 27;9:e95418. doi: 10.2196/95418 (PMC13712171; doi:10.2196/95418)
Supplement: Supplementary file 5 [file nursing-v9-e95418-s005.docx]

**Multimedia Appendix 5: Participant characteristics**

### Table 1: Participating community pediatric continence clinics

| **Site ID** | **Location in England** | **Participant ID** |
| --- | --- | --- |
| Site A | South-East | 1, 2, 3 |
| Site B | South-East | 4, 5, 6 |
| Site C | North-West | 7, 8, 20* |
| Site D | South-East | Participants 9, 10**, 11, 12 |
| Site E | South-West | Participants 13, 14, 15, 16, 17, 18, 19 |

**Participant 20 participated in a focus group discussion but did not complete NoMAD questionnaires*

***Participant 10 completed NoMAD questionnaires but did not participate in a focus group/interview*

### Table 2: NoMAD questionnaire sample N=19

| **How would you describe your professional job category?** | **Baseline (%)** |
| --- | --- |
| Specialist pediatric continence nurse (Band 6 or above) | 14 (73.7) |
| Pediatric continence nurse / non-nurse (Band 5 or below) | 5 (26.3) |
| **How many years have you worked in a pediatric continence clinic setting?** |  |
| Less than one year | 4 (21.0) |
| 1-2 years | 3 (15.8) |
| 3-5 years | 5 (26.3) |
| 6-10 years | 4 (21.0) |
| 11-15 years | 1 (5.3) |
| More than 15 years | 2 (10.5) |

**Band 5 nurses may be newly qualified or staff nurses. Band 6 nurses are who deliver advanced clinical assessments and may lead a team.*

### Table 3: NoMAD questionnaire: Use of URApp at baseline and final follow up* N=19

| **Do you currently use URApp with patients in your clinic?** | **Baseline** | **Final follow-up** |
| --- | --- | --- |
| Never use | 10 | 3 |
| Use sometimes | 8 | 15 |
| Use routinely | 1 | 1 |

**Final follow-up; Site A-D 18 weeks, Site E 12 weeks*
