# Supplementary material for: Process Evaluation of a Digital Smartphone Intervention (URApp) to Support Adherence to Bladder Training in Community-Based Specialist Pediatric Continence Care in England: Mixed Methods Study
Source: JMIR Nurs. 2026 Oct 27;9:e95418. doi: 10.2196/95418 (PMC13712171; doi:10.2196/95418)
Supplement: Supplementary file 6 [file nursing-v9-e95418-s006.docx]

**Multimedia Appendix 6: NoMAD questionnaire data: Descriptive analysis**

| **Team median construct and sub/construct scores** | **Descriptive analysis** |
| --- | --- |
| Agreement (high scoring items) | NoMAD questionnaire responses indicated that teams were largely positive in relation to URApp, they had a good understanding and opinion of the intervention, with high agreement in relation to ‘coherence’ items from baseline, through to the 18-week timepoint. Sites A and E* agreed in relation to all ‘coherence’ items at all timepoints, and Sites B, C and D showed agreement at the 18-week point. This suggests good understanding of how using URApp differed from existing care pathways, and the potential value of URApp in relation to day-to-day practice. At the 18 week point sites A-D agreed that they were open to working with colleagues in new ways to use URApp and would continue to support URApp (cognitive participation). All but one site (C) agreed that there were enough resources to support URApp, (collective action, item 14) and all agreed that feedback about URApp could be used to improve it in the future (reflective monitoring, item 19). Sites A-D all agreed that they could modify how they worked with URApp (reflective monitoring, item 20) at the 18-week point. |
| Neutrality (neither agree nor disagree) | Participants struggled to fully engage with URApp and put it into operation in their day-to-day practice, (collective action) and they also found appraisal of URApp challenging (reflexive monitoring). Sites A, C and D were still reporting neutrality in relation to nine sub-constructs at the 18 week point; 5.initiation - Site D, 6.Legitimation - Site A, 9.interactional workability – Sites A&D, 10.relational integration - Site D, 12/13.skill set workability - Site A&C, 15.contextual integration – Sites A&D, 16.systematisation - Site D, 17.communal appraisal - Sites A&D, and 18.individual appraisal - Sites A&D. This suggests that participants may not have had enough meaningful opportunities to interact with patients who were using URApp to enable them to form either a positive or negative opinion of the intervention. Site E did not complete questionnaires at the 18-week timepoint, but 12-week data conveyed neutrality in relation to six sub-constructs (6. legitimation, 12. skill set workability, 16. systematisation, 17. communal appraisal, 18. individual appraisal and 20. reconfiguration). |
| Disagreement (low scoring items) | Only two sites (A&C) disagreed in relation to three sub-constructs at the 18-week point (11. relational integration - Site A, 14. contextual integration – Site C, 16. systematisation - Site C). This suggests more negative perceptions at these two sites in relation to how confident participants were in other people's ability to use URApp, insufficient resources being available to support URApp, and a lack of awareness in relation to reports about the effects of URApp. Low scoring sub-constructs were located within the ‘collective action’ and ‘reflexive monitoring’ constructs, again suggesting issues with implementation and appraisal of the intervention. It also became apparent that different meaning had been assigned to NoMAD item 11, I have confidence in other people's ability to use URApp. In one site this item was interpreted as referring to colleagues’ ability, whereas in another it was interpreted as having confidence in young people’s ability to use URApp. |
| **Final follow-up Site E 12-weeks* | |

**NoMAD questionnaire: 4.** **Please describe how/who you sometimes use URApp with (free text)**

**Baseline**

Site A:

For teenagers to manage their input/output.

Older children/teenagers to help with drink reminders, and to support compliance with completing bladder/bowel diaries.

Teenage patients or those who have a particular interest in technology.

Site B:

Use for children with voiding postponement to encourage toileting routines and regular fluid intake.

Site C:

Offer patients with daytime bladder issues to record fluid intake and toileting.

Site E:

To ask children to record drinking and wetting to show me.

I have signposted them to the app if they are struggling with motivation – compliance.

**6 Weeks**

Site A:

Children with a phone who have anxiety about completing paper forms or talking about their bladder and bowel symptoms.

Older children/teenagers who are struggling to keep up with fluid intake targets and need support.

Site B:

Older children.

I inform clients about it and show them the app, to support bladder training advice and try to improve compliance.

Use for voiding postponement and regular fluid intake with use of alarms.

Site C:

Advise patients to download and use for review at next appointment.

Daytime wetting. Encourage regular drinking and toileting.

Site D:

Teenage patients with day wetting

At present trialling it but no patient has yet to start using it.

Site E:

Teenagers to record daytime wetting. To get a diary of what has been happening.

Older children to support with bladder training.

I have shown them the APP and suggested it would a useful tool to help them keep a more detailed Diary of their continence - and to show me next time we meet.

Patients who forget to drink regularly and those who are recording drinking / toileting.

Usually discuss with teenagers.

**12 Weeks**

Site A:

Advise to give to patients for drinks/ toileting reminders, and to collate urine output information.

Site B:

I will show the app to anyone I feel would benefit from using and is engaged in their treatment. To help them take responsibility for remembering to visit the toilet regularly and to drink regularly.

Use for children with voiding postponement to encourage toileting and regular fluid intake.

Site C:

Offer to patients to record information.

Dependant on history and referral.

Site D:

Teenage patients with day wetting.

Patients that attend and appear appropriate to use with and are interested.

I have asked a few teenage patients to use but not had engagement from those. Currently waiting to follow up with a young person who struggles to engage with me at all or give me details so have asked them to use app and share information with me.

Site E:

With patients to support with Bladder training.

With patients who struggle to remember when to drink so they can log it and to help track progress/changes with bowel/bladder.

Sign post to APP age appropriate.

**18 Weeks**

Site A:

Secondary school patients.

Site B:

Teenagers or older children.

Young people with wetting difficulties, as a reminder and record of voiding and drinking habits. The young person does need to be motivated.

Children who have voiding postponement to encourage routines and regular fluid intake improve monitoring.

Site C:

Daytime wetting. With young people who have a phone.

Site D:

patients with day wetting.

Teenage patients.

**Raw data: NoMAD median scores by site - Part B: Questions 1-20**

| **SITE A** |  |  |  |  |  |  |  |
| --- | --- | --- | --- | --- | --- | --- | --- |
| **Coherence** | Q1 | Q2 | Q3 | Q4 |  |  |  |
| baseline | 4.00 | 4.00 | 4.00 | 4.00 |  |  |  |
| 6 weeks | 4.00 | 4.00 | 4.00 | 4.00 |  |  |  |
| 12 weeks | 4.00 | 4.00 | 4.00 | 4.00 |  |  |  |
| 18 weeks | 4.00 | 4.00 | 4.00 | 4.00 |  |  |  |
| **Cognitive Participation** | Q5 | Q6 | Q7 | Q8 |  |  |  |
| baseline | 3.00 | 3.00 | 4.00 | 4.00 |  |  |  |
| 6 weeks | 4.00 | 3.50 | 4.00 | 4.00 |  |  |  |
| 12 weeks | 3.50 | 3.50 | 4.00 | 4.00 |  |  |  |
| 18 weeks | 4.00 | 3.00 | 4.00 | 4.00 |  |  |  |
| **Collective Action** | Q9 | Q10 | Q11 | Q12 | Q13 | Q14 | Q15 |
| baseline | 4.00 | 4.00 | 3.00 | 4.00 | 3.00 | 4.00 | 4.00 |
| 6 weeks | 4.00 | 4.00 | 3.50 | 3.50 | 3.00 | 3.00 | 3.50 |
| 12 weeks | 4.00 | 4.00 | 3.50 | 3.50 | 3.00 | 3.00 | 3.50 |
| 18 weeks | 3.00 | 4.00 | 2.00 | 3.00 | 4.00 | 4.00 | 3.00 |
| **Reflexive Monitoring** | Q16 | Q17 | Q18 | Q19 | Q20 |  |  |
| baseline | 4.00 | 4.00 | 3.00 | 4.00 | 4.00 |  |  |
| 6 weeks | 3.50 | 4.00 | 4.00 | 4.00 | 3.50 |  |  |
| 12 weeks | 3.00 | 4.00 | 4.00 | 4.00 | 3.50 |  |  |
| 18 weeks | 4.00 | 3.00 | 3.00 | 4.00 | 4.00 |  |  |

| **SITE B** |  |  |  |  |  |  |  |
| --- | --- | --- | --- | --- | --- | --- | --- |
| **Coherence** | Q1 | Q2 | Q3 | Q4 |  |  |  |
| baseline | 4.00 | 3.00 | 3.00 | 4.00 |  |  |  |
| 6 weeks | 4.00 | 3.00 | 4.00 | 4.00 |  |  |  |
| 12 weeks | 4.00 | 4.00 | 4.00 | 4.00 |  |  |  |
| 18 weeks | 4.00 | 4.00 | 4.00 | 4.00 |  |  |  |
| **Cognitive Participation** | Q5 | Q6 | Q7 | Q8 |  |  |  |
| baseline | 3.00 | 3.00 | 4.00 | 4.00 |  |  |  |
| 6 weeks | 4.00 | 4.00 | 4.00 | 4.00 |  |  |  |
| 12 weeks | 4.00 | 4.00 | 4.00 | 4.00 |  |  |  |
| 18 weeks | 4.00 | 4.00 | 4.00 | 4.00 |  |  |  |
| **Collective Action** | Q9 | Q10 | Q11 | Q12 | Q13 | Q14 | Q15 |
| baseline | 4.00 | 3.00 | 3.00 | 3.00 | 4.00 | 4.00 | 4.00 |
| 6 weeks | 4.00 | 4.00 | 3.00 | 3.00 | 4.00 | 4.00 | 3.00 |
| 12 weeks | 4.00 | 4.00 | 4.00 | 3.00 | 4.00 | 4.00 | 4.00 |
| 18 weeks | 4.00 | 4.00 | 4.00 | 4.00 | 4.00 | 4.00 | 4.00 |
| **Reflexive Monitoring** | Q16 | Q17 | Q18 | Q19 | Q20 |  |  |
| baseline | 3.00 | 3.00 | 3.00 | 4.00 | 4.00 |  |  |
| 6 weeks | 3.00 | 4.00 | 4.00 | 4.00 | 4.00 |  |  |
| 12 weeks | 4.00 | 3.00 | 4.00 | 4.00 | 4.00 |  |  |
| 18 weeks | 4.00 | 4.00 | 4.00 | 4.00 | 4.00 |  |  |

| **SITE C** |  |  |  |  |  |  |  |
| --- | --- | --- | --- | --- | --- | --- | --- |
| **Coherence** | Q1 | Q2 | Q3 | Q4 |  |  |  |
| baseline | 3.50 | 4.00 | 4.00 | 4.00 |  |  |  |
| 6 weeks | 4.00 | 4.00 | 4.00 | 4.50 |  |  |  |
| 12 weeks | 3.50 | 4.00 | 4.00 | 4.50 |  |  |  |
| 18 weeks | 4.00 | 4.00 | 4.00 | 4.00 |  |  |  |
| **Cognitive Participation** | Q5 | Q6 | Q7 | Q8 |  |  |  |
| baseline | 3.50 | 3.50 | 4.00 | 4.00 |  |  |  |
| 6 weeks | 4.00 | 4.00 | 4.00 | 4.00 |  |  |  |
| 12 weeks | 4.00 | 4.00 | 4.50 | 4.50 |  |  |  |
| 18 weeks | 4.00 | 4.00 | 4.00 | 4.00 |  |  |  |
| **Collective Action** | Q9 | Q10 | Q11 | Q12 | Q13 | Q14 | Q15 |
| baseline | 4.00 | 3.50 | 3.50 | 3.50 | 3.00 | 3.00 | 3.50 |
| 6 weeks | 4.00 | 4.00 | 4.00 | 3.50 | 2.50 | 2.50 | 4.50 |
| 12 weeks | 4.50 | 4.50 | 4.00 | 4.00 | 2.50 | 2.50 | 4.50 |
| 18 weeks | 4.00 | 4.00 | 3.50 | 3.50 | 3.00 | 2.50 | 3.50 |
| **Reflexive Monitoring** | Q16 | Q17 | Q18 | Q19 | Q20 |  |  |
| baseline | 3.00 | 3.50 | 3.50 | 4.00 | 3.50 |  |  |
| 6 weeks | 2.50 | 3.50 | 4.00 | 4.50 | 4.50 |  |  |
| 12 weeks | 2.50 | 3.50 | 3.50 | 4.50 | 4.00 |  |  |
| 18 weeks | 2.50 | 3.50 | 3.50 | 4.00 | 4.00 |  |  |

| **SITE D** |  |  |  |  |  |  |  |
| --- | --- | --- | --- | --- | --- | --- | --- |
| **Coherence** | Q1 | Q2 | Q3 | Q4 |  |  |  |
| baseline | 4.00 | 3.50 | 3.00 | 4.00 |  |  |  |
| 6 weeks | 4.00 | 4.00 | 4.00 | 4.00 |  |  |  |
| 12 weeks | 3.50 | 3.50 | 4.00 | 4.00 |  |  |  |
| 18 weeks | 4.00 | 4.00 | 4.00 | 4.00 |  |  |  |
| **Cognitive Participation** | Q5 | Q6 | Q7 | Q8 |  |  |  |
| baseline | 3.00 | 3.00 | 4.00 | 4.00 |  |  |  |
| 6 weeks | 3.00 | 3.00 | 3.50 | 3.50 |  |  |  |
| 12 weeks | 3.50 | 3.50 | 4.00 | 4.00 |  |  |  |
| 18 weeks | 3.00 | 4.00 | 4.00 | 4.00 |  |  |  |
| **Collective Action** | Q9 | Q10 | Q11 | Q12 | Q13 | Q14 | Q15 |
| baseline | 3.00 | 3.50 | 3.00 | 3.00 | 3.00 | 3.00 | 3.50 |
| 6 weeks | 3.00 | 3.50 | 2.50 | 3.50 | 2.50 | 2.50 | 3.50 |
| 12 weeks | 3.50 | 3.50 | 3.50 | 3.00 | 3.50 | 4.00 | 4.00 |
| 18 weeks | 3.00 | 3.00 | 4.00 | 4.00 | 4.00 | 4.00 | 3.00 |
| **Reflexive Monitoring** | Q16 | Q17 | Q18 | Q19 | Q20 |  |  |
| baseline | 3.00 | 3.00 | 3.00 | 4.00 | 3.00 |  |  |
| 6 weeks | 2.50 | 3.00 | 3.00 | 3.50 | 3.00 |  |  |
| 12 weeks | 3.00 | 3.50 | 3.00 | 4.00 | 3.00 |  |  |
| 18 weeks | 3.00 | 3.00 | 3.00 | 4.00 | 4.00 |  |  |

| **SITE E** |  |  |  |  |  |  |  |
| --- | --- | --- | --- | --- | --- | --- | --- |
| **Coherence** | Q1 | Q2 | Q3 | Q4 |  |  |  |
| baseline | 4.00 | 4.00 | 4.00 | 4.00 |  |  |  |
| 6 weeks | 4.00 | 4.00 | 4.00 | 4.00 |  |  |  |
| 12 weeks | 4.00 | 4.00 | 4.00 | 4.00 |  |  |  |
| **Cognitive Participation** | Q5 | Q6 | Q7 | Q8 |  |  |  |
| baseline | 3.00 | 3.00 | 4.00 | 4.00 |  |  |  |
| 6 weeks | 4.00 | 3.50 | 4.00 | 4.00 |  |  |  |
| 12 weeks | 4.00 | 3.00 | 4.00 | 4.00 |  |  |  |
| **Collective Action** | Q9 | Q10 | Q11 | Q12 | Q13 | Q14 | Q15 |
| baseline | 4.00 | 4.00 | 4.00 | 3.00 | 4.00 | 4.00 | 4.00 |
| 6 weeks | 4.00 | 4.00 | 4.00 | 3.00 | 3.50 | 4.00 | 4.00 |
| 12 weeks | 4.00 | 4.00 | 4.00 | 3.00 | 4.00 | 4.00 | 4.00 |
| **Reflexive Monitoring** | Q16 | Q17 | Q18 | Q19 | Q20 |  |  |
| baseline | 3.00 | 3.00 | 3.00 | 4.00 | 3.00 |  |  |
| 6 weeks | 3.50 | 3.50 | 3.00 | 4.00 | 4.00 |  |  |
| 12 weeks | 3.00 | 3.00 | 3.00 | 4.00 | 3.00 |  |  |

**NoMAD scores by participant (N=19) /timepoint**

**Site A**

**Site B**

**Site C**

**Site D**

**Site E**
