# Supplementary material for: Process Evaluation of a Digital Smartphone Intervention (URApp) to Support Adherence to Bladder Training in Community-Based Specialist Pediatric Continence Care in England: Mixed Methods Study
Source: JMIR Nurs. 2026 Oct 27;9:e95418. doi: 10.2196/95418 (PMC13712171; doi:10.2196/95418)
Supplement: Supplementary file 7 [file nursing-v9-e95418-s007.docx]

**Multimedia Appendix 7**

**Illustrative themes, corresponding NPT constructs, and quotes (N=19)**

| **Theme 1: URApp has potential (Coherence)** | |
| --- | --- |
| **Sub-theme (sub-construct)** | **Supporting quotation** |
| Prior knowledge of URApp | *I knew there were apps available… but I hadn't actually used one …and I didn't think they were for children, I think that's why I felt they were more for an adult… (Participant 5, Site B-Interview*)* |
|  | *I hadn’t heard of it (Participant 18, Site E)* |
| Positive view of app functionality  (internalisation) | *I have dummy run it myself, and there’s really good things to it. It’s a really good app (Participant 3, Site A)* |
| Positive view of app as age appropriate  (internalisation) | *it is nice to have something for them I would say, because with all the toileting information, well a lot of it, is based on younger children in a way, and I felt that this was something a bit more relatable to them... it’s private and it's something you can do so you don't have to keep saying to mum or dad, this is what's happened, or I've wee’ed this many times, you know, it's just it's a bit more private for them and I just think it's relatable to them really. (Participant 4, Site B-Interview*)* |
| To support diagnosis  (communal specification) | *I suppose you’re almost then looking at URApp and working in two different ways. You’re partly using it as a diagnostic [to record input/output]…. (Participant 2, Site A)* |
| To aid ongoing adherence to bladder training (communal specification) | *…and then partly using it as treatment implementation [reminders to drink go to toilet]. (Participant 2, Site A)* |
| Paper bladder diaries not returned (differentiation) | *[bladder diaries] very few and far between they get returned as well, so compliance is an issue with completing charts. That’s why we thought the app would be a bit easier, because they’re constantly on the phone, even parents. (Participant 7, Site C)* |
| Forgetting to introduce URApp  (differentiation) | *...as long as we remember, because it’s fairly new to us still, and you have your little script don’t you that you go through in your mind? (Participant 12, Site D)* |
| Can be used for bowel issues  (individual specification) | *It probably is wider ways, isn't there? I mean, I know it's got the stool bit on there as well, hasn’t it, bowels... So that was quite useful and I guess we can introduce it just for the bowels as well. (Participant 6, Site B-Interview*)* |
| Can be used with young people with overactive bladder  (individual specification) | *it's good for the children with the overactive bladders as well, because they forget to drink and because they're always responding to their bladder signals they don't think to actively think to go to the toilet. And sometimes I say instead of, I know you're going a lot, but sometimes it's nice to actively take control of your bladder and think to go. To try and re-train (Participant 5, Site B-Interview*)* |
| URApp could be used in place of a wobble watch  (communal specification) | *...the reminder side of things on [URApp] we did discuss that and found that that might be something because quite often we say have a wobble watch... the reminders might be helpful in that situation, with the ADHD ones, definitely. (Participant 6, Site B-Interview*)* |
| Recording additional symptoms via the daily diary function  (interactional workability) | *Because she had been writing notes about... so she would record if she had damp pants, and whether that was happening on the way to the toilet or when she’d finished, but she was also recording things like she had tummy ache, and that because she would say it hurts, and I had to expand on that to say when you say it hurts what do you mean, is it stinging, is it tummy ache, what sort of pain are we talking about? But she’d written in the notes bit what was happening when she was going to the toilet, and I thought oh if she could print that off so that I could see that in a printout that would be really useful to have that information (Participant 9, Site D)* |
| URApp encouraging young people to develop their awareness of drinking/using the toilet  (individual specification) | *…although she couldn’t use [URApp] in school, because she knew that you can set reminders on the phone for drinking and weeing, it did make her think about that when she was at school, that at breaktimes and lunchtimes she needed to have a drink and go for a wee. So although the phone wasn’t physically with her reminding her, URApp did just make that link with her to say oh yeah I do need to go for a wee because that would have been a reminder if I’d have had it with me. Do you see what I mean? (Participant 9, Site D)* |
| **Theme 2: It's easy to introduce… BUT (Cognitive participation)** | |
| **Sub-theme (sub-construct)** | **Supporting quotation** |
| Engagement in terms of ‘introducing’  URApp  (legitimation) | *I find it's been beneficial really in the ones that I have given it to, but they are all keen and then it's whether it's carried on. But I quite like introducing it because it's something that's different and we haven't tried before… you say about this URApp and they think oh actually that's something good rather than just giving them a bit of paper to fill in. (Participant 6, Site B-Interview*)* |
| Introduce it as something different  (communal specification) | *I think that's one of the things I do like URApp, because you can introduce it as something different, something new, you know, into the mix. You know, we're not just sending them away for three months doing the same old thing and filling the same [paper] charts. (Participant 5, Site B-Interview*)* |
| Easy to introduce  (legitimation) | *I think it’s easy to talk to them about it... in terms of actually putting it out there for them to try it, that’s quite a straightforward thing to do... (Participant 12, Site D)* |
| Would continue to use in future  (activation) | *I'm quite keen on continuing to do it really, and I'm sure over time that will be a way of monitoring maybe and we get a bit more data. (Participant 6, Site B-Interview*)* |
| Signposting to URApp not effective  (enrolment) | *Quite often what I find, if I signpost a young person to it, then they, you see them or talk to them again and they haven't downloaded it or haven't looked at it. (Participant 19, Site E)* |
| Asking young people to download URApp in clinic more effective  (legitimation) | *Most of them we get them to download it in clinic. So we know they’ve got it. (Participant 7, Site C)* |
| Adding URApp link to team templates (activation) | *I've created [links] for everyone to use, and I use them to send it out (Participant 13, Site E)* |
|  | *We could update that [letter], that would be a good way of getting it out there to the new patients. (Participant 9, Site D)* |
| Leaflet with the URApp link and logo  (activation) | *[leaflet] it’s literally just a screenshot of the URApp website printed out, so they know what they’re looking for. So we show them that in clinic, and then they go on the App Store and download it then, and then they have to set the passcode up, and then we go through little bits with it, and then the kids normally will have a little go of it... (Participant 7, Site C)* |
| Limited nurse time to act as a ‘champion’ for URApp  (initiation) | *I've emailed [the team], asked how many they’ve introduced it to. (Participant 6, Site B-Interview*)* |
| Individual (not team) engagement from nursing staff  (initiation) | *...initially we had a team meeting with all of [us] attending one of these virtual sessions at the beginning [site initiation meeting] and then it's been for us individuals to implement it in our own patients... (Participant 18, Site E)* |
| Create a QR code  (enrolment) | *…making posters for in clinic with QR codes on or something, because that’s the attention span we’re getting timewise, scan that’s... rather than going into it, and maybe, I don’t know, maybe a little guide for parents. (Participant 7, Site C)* |
| Animation to facilitate the introduction of URApp (for young people , parents, health professionals)  (enrolment) | *cause a lot of young people, they like YouTube, don't they? You know, most of my children sit on the toilet. TikTok as well. Yes. All these different things that I don't do. They take their phone on the toilet with them. (Participant 5, Site B-Interview*)*  *Just a bit of just a simple... like a presentation on this is... like a PowerPoint, just on the screens, and how to use it, and just a bit more informative really, I felt like I just had to find my own way with it. (Participant 8, Site C)*  *I think [animation] is a starting point for a parent to have a quick look first of all to how you feel about it... We have such a mix of parent and child that we work with, their abilities are so varied, so I think anything that we’ve got as alternatives is better. (Participant 11, Site D)* |
| **Theme 3: Behaviour change is hard (Collective Action)** | |
| **Sub-theme (sub-construct)** | **Supporting quotation** |
| Poor uptake  (relational integration) | *I don’t tend to get the uptake of them wanting to use it (Participant 3, Site A)* |
| Young people still lacked motivation  (relational integration) | *…the actual app is good, it's just getting the young people to use it (Participant 16, Site E)* |
| Lack of long-term engagement  (relational integration) | *she said it was great, but it's tailed off, and this is the trouble. (Participant 5, Site B-Interview*)* |
| Lack of engagement from young people demotivating for staff  (relational integration) | *I'm not seeing the massive change I thought I would see, unfortunately, I'll still be suggesting it. (Participant 15, Site E)* |
| Positive engagement with URApp:  (interactional workability)  Neurodivergent patients who like 'tech'  Rewards – liked by younger children  Access to a smartphone | *[neurodivergent patients] are very good with their phones and things, and they like the routines as well so. And that's the particular boy that done quite well, actually. He's an intelligent neurodiverse one, and he did do very well with it... the neurodiverse [patient] was more open to it and wanted to do it. Yeah, but that's because of the tech type thing. And they love that sort of thing. (Participant 6, Site B-Interview*)*  *…[11yr old] quite like the reward. He was quite chuffed that he'd earn a silver, so yes. (Participant 6, Site B-Interview*)*  *[10yr old] really likes the app, more because it means that she is allowed to use a mobile phone… she did say that she likes using it… [and] found it easy… (Participant 9, Site D)* |
| Lack of motivation/engagement:  (interactional workability)  Age of child/young person a factor  Engagement takes time | *motivation comes later, it doesn’t come with the 11 and 12 year olds (Participant 3, Site A)*  *the older teenagers I just haven't been particularly able to engage, because they've had this problem for so long and they've tried so many things, capturing that activity in an app, I think they struggle to sort of motivate themselves to do it. (Participant 15, Site E)*  *…often it takes a while for parents and children to engage. (Participant 4, Site B-Interview*)* |
| Follow up conducted remotely  (interactional workability) | *if they came to our face-to-face appointment and their fluid intake was really poor, we would possibly advise on URapp. But then the next appointment would be a follow up telephone review with the parents (Participant 16, Site E)* |
| Data capture in URApp by young people (without parental support)  (skill set workability) | *I would have no access to it [URApp], it’s not like it would be shared to me, the reliance is on them (Participant 3, Site A)* |
| Parents/carers support and manage their child’s bladder training  (skill set workability) | *if you’ve got the young person directly collecting the data how is the parent or carer going to monitor that... (Participant 2, Site A)* |
| Parents/carers key to recording fluid input/output  (skill set workability) | *So it [URApp] saves them doing paper diaries, which they don’t really do anyway. …for a paper diary to be filled in we really have to push and be on the parents. We have to ring them, “Have you done that?” ...you have to check in with them all the time, just to get even two to three days’ worth of data off them. (Participant 7, Site C)* |
| Allowing child/young person to have ownership  (skill set workability) | *for the parents to be able to take that step back and not be on their back so much, and we do say that, just take a break from it, set a reminder and it's them to listen to that reminder rather than you saying off you go to the toilet, have you been, look at the time, when did you last go. Take that step back, take the responsibility off and let the child. (Participant 6, Site B-Interview*)* |
| Asking young people to remind and show them how they had been using URApp  (skill set workability) | *when I’ve asked them to use it, I've asked them to remind me and show me [at their next appointment], the phones. None of them have reminded me, and then they've left and I thought I needed to ask them. That's kind of a recurring pattern when it's kind of busy in clinic really. (Participant 13, Site E)* |
| Parent views on medication being a better option than bladder training (skill set workability) | *They don't see [bladder training] as a way of getting the problem sorted (Participant 15 Site E)*  *[parents] Nag for the medicines, they will all... they won’t opt for URApp. (Participant 2, Site A)* |
| Joint effort – child and parent  (relational integration) | *...it’s very much joint effort, because you do the clinic appointments with these children with a parent... sometimes it might be with a child alone, but the conversation is very much with the child, but also with the input from the family, from the parent that’s with them. We are really focused on it being the child, but there’s things they can’t answer, and the parents will often say, “I have to nag them to do the drinking, it’s they won’t do the drinking,” or whatever it might be, or, “I have to do the reminding to use the toilet, whatever.” So again their parents are so involved, they have to be with these children. (Participant 11, Site D)* |
| Pro-active patients may be more motivated to engage in research co-production  (relational integration) | *I wonder if some of the young people that were involved in creating [URApp] would have had that independent streak in the first place, because if you care enough about your current condition that you want to get involved in a research project and you want to try a new thing, they’ve already got that internal motivation, that internal independence, whereas actually what we find, not with all of our teenagers. (Participant 2, Site A)* |
| Pro-active patients make quicker progress  (relational integration) | *things don't always get better until they do take that control (Participant 6, Site B-Interview*)* |
| Lack of confidence in changing from paper to electronic bladder diaries  (contextual integration) | *…other concern from parents and carers would be that actually if you’ve got the young person directly collecting the data how is the parent or carer going to monitor that. Whereas when they’ve got a paper diary invariably it goes up on the fridge, or it gets stuck up in their bedroom (Participant 2, Site A)*  *part of me obviously wants to move with the times and everything else, but actually as a parent of teenagers would I want them doing it on an app? Probably not, no, not without being able to... because you don’t know... then you’re obviously constantly, “Have you done it? Did you do your app today? Have you filled it in today?” That would cause problems in my home for me continuously having to ask… (Participant 3, Site A)*  *I don't know if I'd be confident to use URApp just as it is without the charts [paper bladder diaries], but I think that's just my confidence in it, and getting that information, and knowing where I could put that information because with the charts it goes on to their records… the apps a really good way of reminding them with their routines, the toilet, things like that, and looking a bit more at what's happening, how much they're drinking. So I'd quite like the charts at this moment in time for a short study and then the app to be used as a longer process for them, I think. Possibly, until I'm a bit more confident with it, perhaps. (Participant 4, Site B)* |
| Shared parent/child login, or secondary parent/carer URApp platform  (contextual integration) | *we do get a lot of families that they’ll say, “I’ve lost the piece of paper, but I’ve written it out on my phone.” So then they come to clinic and they’re showing you their phone, and you’re having to write it out onto paper. Whereas if it could be emailed, like I said emailed in, or we could print it or something, but obviously if [data] was in an app instead that the parents had as well as the child, they could be recording that info there as well (Participant 11, Site D)* |
| Data export function from URApp not used  (contextual integration) | *...they showed me on [their] phone... that they’d been writing this stuff down, but obviously because it’s on their phone I can’t then put it onto her record, because it’s on their phone. So if there was a way to actually print that or email that to me to say this is what was happening on these days, that would have been quite useful information to have. (Participant 9, Site D)*  *...you don’t get... I’ve never been presented with lovely data [laughter]. (Participant 3, Site A)* |
| Timing of NoMAD questionnaires | *...we might not review as early as this study needs us to do that if that makes sense. [Researcher: How regularly do you think you see them?] it depends on their issues... I would say every three to four months. (Participant 4, Site B-Interview*)* |
| **Theme 4: Using URApp in a school environment (Reflexive Monitoring)** | |
| **Sub-theme (sub-construct)** | **Supporting quotation** |
| Impact of school culture on engagement with bladder training (systematization) | *the children that go to the really good schools that support them with the plans are the children that make the progress, the children that aren't supported at school don't make that progress (Participant 13, Site E)* |
|  | *there are quite a lot of barriers to doing the good toileting and the good drinking in school (Participant 9, Site D)* |
| Lack of toilet access  (systematization) | *Massive issues over toilet access in the school, and drinking. If the education sector got that on-board we probably wouldn’t have a job… they could probably use their phone more than they’re allowed to go to the toilet. (Participant 7, Site C)* |
| Stigma - Using a toilet/medical pass to leave lessons to use the toilet  (communal appraisal) | *some of them will get a toilet pass, but even with a toilet pass a) they don’t want to use it because they don’t want to appear different to their friends, so they don’t actually want to go out of the classroom… (Participant 3, Site A)*  *she [patient] just said I'm not going to [use a medical pass] … She's only got a few months left of school, so hopefully it's not going to be too impactful for her for too much longer… (Participant 17, Site E)* |
| Not able to access phone at school  (communal appraisal) | *Then there’s been at least four that I’ve spoken to about it, and the person’s just gone, “Oh they’re not allowed their mobile phones, they’re not allowed the phones in school.” when they can’t use it in school they’re very much like no, can’t use this phone, and a little bit dismissive about it. (Participant 11, Site D)*  *the other teenage girl, she was quick to say I can't have it in school, that's it. Job done. (Participant 6, Site B-Interview*)* |
| Modifying how worked with URApp in future  (reconfiguration) | *There is a use for URApp, because if they input the information and export it... we can just say, ‘Let’s export your information in clinic’, and then we get a view of what’s been going on, rather than, ‘I’ve left [paper bladder diary] in the car’, or… ‘I left it on the table’. It’s easier, because it’s on the phone in clinic. So there is a use for it (Participant 8, Site C)*  *I could see it working [in future, in relation to patient-initiated follow-up] because yes, we'll send them away with URApp and saying this is what you can be working on, putting it all on here and giving them this period of time to do it all in. (Participant 5, Site B-Interview*)* |
| *Alternatives to using URApp in the school environment - using URApp at weekends*  (reconfiguration) | *…the fact that you can change [reminders] for weekdays and weekends, I’ve then actually used that to say, “Well, if you’re managing to finish your water in the school day let’s set up the reminders at the weekend to remind that.” And I think I’ve had more success with that rather than actually collecting the actual data itself, does that make sense? (Participant 2, Site A)* |
| More usable bladder diary data with URApp  (individual appraisal) | *that [neurodivergent male] I said, you know, normally you sit here and you shrug and you don't know, you don't know. But we've got that phone now, and I've seen more data now than I ever have done. I was surprised he was doing it. So he wasn't one I thought, actually, you're going to just download it and off you're going to go because that's normally what happens. But now I bought him back sort of the, I think two months later. Yeah. and we've got some data. (Participant 6, Site B-Interview*)* |
| **Indicates interview, all other participants took part in focus groups* | |
